# Polyphenolic Diversity, Antioxidant Activity, and Anticancer Potential of *Celtis australis* L. Fruits: New Insights

**DOI:** 10.1002/fsn3.71800

**Published:** 2026-04-19

**Authors:** Jihane Touhtouh, Tarik Aanniz, Abdelhakim Bouyahya, Laura Acquaticci, Ghazal Namazzadeh, Giovanni Caprioli, Waleed Al Abdulmonem, Mohammed Alorini, Taoufiq Benali, Khalil Hammani

**Affiliations:** ^1^ Laboratory of Natural Resources and Environment, Polydisciplinary Faculty of Taza Sidi Mohamed Ben Abdellah University of Fez Taza Morocco; ^2^ Biotechnology Laboratory (MedBiotech), Bioinova Research Center, Rabat Medical and Pharmacy School Mohammed V University Rabat Morocco; ^3^ Laboratory of Human Pathologies Biology, Department of Biology, Faculty of Sciences Mohammed V University in Rabat Rabat Morocco; ^4^ School of Pharmacy, Chemistry Interdisciplinary Project (ChIP) University of Camerino Camerino Italy; ^5^ Department of Pathology, College of Medicine Qassim University Buraydah Saudi Arabia; ^6^ Laboratory of Ecotoxicology, Bioresources, and Coastal Geomorphology, Polydisciplinary Faculty of Safi Cadi Ayyad University Safi Morocco

**Keywords:** anticancer activity, antioxidant activity, *Celtis australis* L, Morocco, nutraceuticals

## Abstract

*Celtis australis* L. was selected for investigation due to its traditional medicinal use and the limited number of studies available, particularly concerning its anticancer potential. This study examined the phenolic composition, the antioxidant activity, and the anticancer effects of methanolic, ethanolic, and aqueous extracts of *C. australis* L. The in vitro cytotoxic efficacy was assessed with the MTT assay on six tumor cell lines (HeLa, MV4‐11, OCI‐AML3, DAOY, OSN‐76, and U‐138‐MG) and two non‐cancerous cell lines (HEK293T and hCMEC/D3). *C. australis* L. extracts exhibited moderate antioxidant activity, as evidenced by their high IC_50_ values (110–436 μg/mL). The FRAP reducing power was low (2.09–6.24 mg AAE/g), and the aqueous extract was least active, with IC_50_ values ranging from 152 to 207 μg/mL in the ABTS assay. The HPLC‐ESI‐MS/MS analysis indicated that the extracts contain a variety of phenolic compounds dominated by neochlorogenic acid, chlorogenic acid, procyanidin B2, (−)‐epicatechin, isoquercitrin, rutin, phloretin, and delphindin‐3,5‐diglucoside. Cytotoxicity assays demonstrated a dose‐dependent reduction in cell viability, with DAOY cells exhibiting the highest sensitivity. Water extract exhibited significant inhibitory effects at elevated concentrations, whereas ethanol extract showed greater potency at intermediate doses (7.5%–50%) for specific cancer cell lines. Methanol extract exhibited a more gradual impact and showed reduced potency at elevated doses. According to IC_50_ values, the ethanolic extract was the most efficacious in four out of the six cancer cell lines, whereas water extract demonstrated notable potency for OCI‐AML3 and OSN‐76 but were less effective on normal cells, suggesting selectivity based on cell type. The selectivity index indicated that methanol exhibited the highest selectivity, especially against OSN‐76, MV4‐11, OCI‐AML3, and HeLa cell lines, implying its predilection for cancer cells over normal cells. Ethanol demonstrated notable selectivity, particularly toward OSN‐76 and MV4‐11. Conversely, water extracts exhibited minimal selectivity across all strains. In summary, methanol and ethanol extracts demonstrate the best potential for selective anticancer efficacy.

## Graphical abstract

Extracts from *Celtis australis* fruit exhibit good antioxidant activity—Extracts from *C. australis* fruit inhibit selectively the development of various human cancer cell lines in a dose‐dependent manner. Extracts from *C. australis* fruit are rich in phenolic compounds mostly with known anticancer activity.

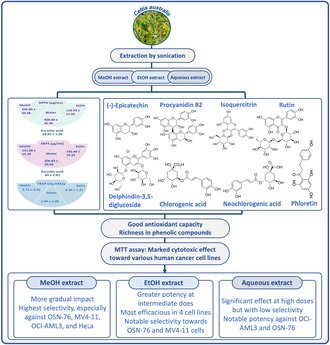

## 1 Introduction

According to the World Health Organization (WHO), cancer is the second leading cause of death globally, accounting for an estimated 10 million deaths in 2020 and representing approximately 1 in 6 deaths worldwide (Ferlay et al. 2021). Despite advancements in cancer treatment, therapeutic responses are still inadequate, and side effects are prevalent in most cases (Bakrim et al. 2022; Rezvan et al. 2025). The concept of cancer prevention has gained much attention in recent years with epidemiological data showing that dietary patterns play a critical role in disease risk, with approximately 90%–95% of cancers linked to lifestyle and environmental factors and nearly 30% directly attributable to nutrition (Nasir et al. 2020). The PREDIMED trial, involving 7447 participants (55 to 80 years of age, 57% women), showed that following a Mediterranean diet rich in olive oil or nuts considerably reduces major cardiovascular risk by ~30% and may also contribute to cancer prevention likely due to its anti‐inflammatory, antioxidant, and metabolic‐regulating effects (Guasch‐Ferré et al. 2017). Consistent with these findings, a higher consumption of fruits, vegetables, and spices has been associated with reduced incidence of stomach cancer by 40%–60% in Italy compared with those with the lowest intake (Buiatti et al. 1989), while India's comparatively low prevalence of colorectal cancer, 3–5 times lower than in Western countries, has been partially attributed to elevated consumption of spices, such as turmeric, with curcumin intake estimated at 100–200 mg/day in traditional Indian diets (Giles et al. 2023; Kaefer and Milner 2008). Other epidemiological studies indicate that communities adhering to plant‐based diets exhibit a reduced incidence of stomach cancer, hence reinforcing the significance of plant‐derived chemicals in chemoprevention (Aleebrahim‐Dehkordy et al. 2017; Trojan‐Rodrigues et al. 2012). Multiple studies and clinical trials linked food with the risk and progression of cancers such as colon, breast, and prostate, hence strengthening the notion of diet‐related malignancies (Divella et al. 2020). Recent attention has been directed toward natural chemicals obtained from medicinal plants, which display various pharmacological activities, with numerous plant extracts showing chemopreventive effects (Aleebrahim‐Dehkordy et al. 2017; Al‐Hwaiti et al. 2021; Anggraini et al. 2026; Castañeda‐Espinoza et al. 2026; Chien et al. 2021; Guldar et al. 2025; Javrushyan et al. 2026; Kagawa et al. 2019; Rezvan et al. 2025; Shahbaz et al. 2024; Zeppa et al. 2025). Hence, vegetables and fruits serve a dual function in oncology as sources of nutraceuticals. In cancer prevention, they can significantly reduce the risk of cancer through healthy dietary habits. In therapy and survivorship, they can improve treatment tolerance, reduce adverse effects, enhance quality of life, and potentially reduce the risk of recurrence. Nutrients are also a source of biocompounds that could serve as scaffolds for cancer drugs and epidrugs (Bakrim et al. 2025).

The genus *Celtis* (*Cannabaceae* Martinov) has approximately 73 species, which are prevalent in temperate regions of the northern hemisphere and certain areas of central/north Africa and South America (Hwang 2003; Martins et al. 2015; Yang et al. 2013). *Celtis australis* L., generally referred to as the hackberry, is one of the most extensively researched and employed species. This species, indigenous to the Mediterranean region, is prevalent in semi‐arid and temperate sub‐humid bioclimates, especially in Europe and North Africa (Safari et al. 2023). In Morocco, *C. australis* L. is referred to as “*Teghzaz*” (Benamar et al. 2022). It is a deciduous, suckering tree distinguished by its smooth, whitish bark, deciduous foliage, pubescent young branches, and subglobose drupes (Benamar et al. 2022). The hackberry fruits are recognized for their stomachic therapeutic qualities (Benamar et al. 2022) and from a nutritional perspective, they are rich in dietary fiber, phenols, vitamins (tocopherols, carotenoids), and minerals (Ota et al. 2017). Preparations of leaves and fruits exhibit prophylactic efficacy in the management of amenorrhea, colic, diarrhea, dysentery, peptic ulcers, and menstrual hemorrhage (Touhtouh et al. 2025). Traditionally, both leaves and fruits have been utilized for their therapeutic properties, particularly for the treatment of gastrointestinal disorders (diarrhea, dysentery, colic, and peptic ulcers) and for addressing regular or irregular menstrual cycles in women of reproductive age and managing intermenstrual bleeding and colic (Abbouyi et al. 2015; Demır et al. 2002; Filali‐Ansari et al. 2016; Perović Jovana et al. 2023).

Aqueous and organic extracts, together with fractions from different parts of *C. australis* , have exhibited significant antioxidant (Cascales et al. 2021; Hammash et al. 2016; Ota et al. 2017; Safari et al. 2023; Shokrzadeh et al. 2019), antimicrobial potency (Perović et al. 2023) as well as gastroprotective and hepatoprotective properties in vivo (Shokrzadeh et al. 2019). The seeds' ethyl acetate extract from Morocco exhibited significant wound‐healing properties (El Abbouyi et al. 2021). *C. australis* L. extract markedly decreased plasma hepatic enzyme activity and safeguarded mice from liver tissue damage caused by CCl_4_ (Abbouyi et al. 2015; Kumar et al. 2017). Bark and fruit extracts have potent analgesic, anti‐inflammatory, and antinociceptive effects, as well as anticancer activities against some cancer cell lines (El‐Alfy et al. 2011). However, overall, only a limited number of studies focused on the anticancer properties of *C. australis*. Multiple studies on *C. australis* extracts revealed the abundance of phytochemicals, including phenolic compounds, flavonoids, organic acids, and minerals in *C. australis* L. fruits (Touhtouh et al. 2025). Filali‐Ansari et *al*. indicated that flavonoids constitute a significant group in Moroccan *C. australis* L., and their abundance in fruits led to marked anti‐inflammatory, antibacterial, and antioxidant potencies (Filali‐Ansari et al. 2015). Numerous secondary metabolites—including isovitexin, gallocatechin, epigallocatechin, orientin, isoorientin, rutin, quercetin, astragalin, apigetrin, luteolin, apigenin, naringenin, hispidulin, and celtisanin—have been isolated (Badoni et al. 2011; Touhtouh et al. 2025).

*C. australis* L. is historically utilized in Mediterranean folk medicine and is attracting scientific attention due to its pharmacological properties. However, despite the growing interest in plant‐derived anticancer agents, relatively few studies have specifically investigated the anticancer potential of *C. australis* . This gap highlights the need for further research, especially in light of recent literature emphasizing the promising role of bioactive compounds from medicinal plants in cancer prevention and therapy. Hence, we examined in vitro the antioxidant potency and the cytotoxic efficacy of methanolic, ethanolic, and water extracts of *C. australis* L. fruits against a selection of human cancer cell lines compared to non‐cancerous cell lines to assess both potency and selectivity.

## 2 Materials and Methods

### 2.1 Fruits Collection and Extraction

Fruits of *C. australis* L. were collected from the Taza region (northern Morocco) in November 2022 and authenticated at the Scientific Institute, Mohammed V University of Rabat by professor Elkhamar Mohammed, where a voucher specimen was deposited. Dried fruits were ground (0.5–1 mm), and 5 g of powder was extracted with 100 mL of 75% methanol, 75% ethanol, or water using ultrasonic bath (SB‐120DT, China) for 30 min at 25°C–30°C (40 Hz, 120 W) (Guzelmeric et al. 2022). The extracts were filtered using a Buchner funnel and 0.2 mm thick filter paper, followed by the evaporation of solvents using a rotary evaporator at 45°C and 80 mbar pressure. The residual aqueous component was frozen at −20°C overnight and subsequently lyophilized at −55°C at 0.2 mbar pressure. Following the lyophilization process, the extracts were placed in amber bottles and refrigerated at +4°C for the duration of the experiment (Turnalar Ülger et al. 2023).

### 2.2 Antioxidant Activity Assays

The free radical‐scavenging effect of *C. australis* L. extracts was determined using the stable DPPH radical according to Blois (Blois 1958) with some modifications. 1 mL of samples (10 mg/mL) were mixed with 4 mL of methanolic solution of DPPH and incubated; the assay was performed in triplicate, at ambient temperature for 30 min, with ascorbic acid as a positive control. Absorbance was read at 517 nm, and the inhibition IC_50_ (μg/mL) was calculated.

The ABTS cation radical was generated from the reaction between ABTS (7 mM) stock solution and potassium persulfate (2.45 mM) incubated at ambient temperature for 12 h. A volume of 1 mL of samples (10 mg/mL) was mixed with 2 mL of ABTS solution and incubated at ambient temperature for 30 min. The assay was performed in triplicate, with ascorbic acid as a positive control. Absorbance was read at 734 nm, and expressed as IC_50_ (μg/mL) (Re et al. 1999).

The reducing power of the extracts was assessed following the method of Benzie (Benzie and Strain 1996), with some modifications. A reaction mixture containing 1 mL of sample (10 mg/mL), 2.5 mL phosphate buffer (0.2 M, pH 6.6), and 2.5 mL potassium ferricyanide was prepared and incubated at 50°C for 20 min. After adding 2.5 mL of 10% trichloroacetic acid, the mixture was centrifuged (3000 rpm, 10 min). Then, 2.5 mL of the supernatant was mixed with 0.5 mL ferric chloride (0.1%) and 2.5 mL distilled water. Absorbance was measured at 700 nm, the assay was performed in triplicate, and potency was expressed as mg ascorbic acid equivalents per gram of extract (mg AAE/g).

### 2.3 Anticancer Activity

*C. australis* L. extracts were evaluated for their cytotoxic activity on six tumor cell lines: HeLa (Cervical Adenocarcinoma), MV4‐11 and OCI‐AML3 (Acute Monocytic Leukemia), DAOY (Desmoplastic Cerebellar Medulloblastoma), OSN‐76 (medulloblastoma), U‐138‐MG (glioblastoma), and two non‐cancerous cell lines: HEK293T (Human Embryonic Kidney) and hCMEC/D3 (Human Brain Endothelial Cells). Cells were treated with six increasing concentrations (1% to 100% v/v) of methanolic (MeOH), ethanolic (EtOH), and aqueous (W) extracts. Cell viability was then measured using the MTT assay.

#### 2.3.1 Cell Lines and Cell Culture

HeLa (Cervical Adenocarcinoma), MV4‐11, and OCI‐AML3 (Acute Monocytic Leukemia) cell lines were cultured in Roswell Park Memorial Institute 1640 media (RPMI1640; Gibco Life Technologies Corp., USA), supplemented with 10% Fetal Bovine Serum (FBS) (Biowest, France) and 1% penicillin/streptomycin. HEK293T (Human Embryonic Kidney) cells were cultivated in Dulbecco's Modified Eagle Medium devoid of pyruvate (DMEM; Gibco Life Technologies Corp., USA), supplemented with 10% fetal bovine serum and 1% penicillin/streptomycin (Gibco Life Technologies Corp., USA). The DAOY (Desmoplastic Cerebellar Medulloblastoma), OSN‐76 (Human Medulloblastoma), and U‐138‐MG (Human Glioblastoma) cell lines were cultured in DMEM enriched with 10% FBS and 1% penicillin/streptomycin. hCMEC/D3 (Human Brain Endothelial Cells) were cultivated in Endothelial Cell Growth Medium (PromoCell, Germany) augmented with Endothelial Cell Growth Medium Supplement Mix (PromoCell, Germany), which included 0.02 mL/mL fetal calf serum, 0.004 mL/mL endothelial cell growth supplement, 0.1 ng/mL epidermal growth factor, 1 ng/mL basic fibroblast growth factor, 90 μg/mL heparin, and 1 μg/mL hydrocortisone. All cell lines were grown and sustained in accordance with their specific doubling times.

#### 2.3.2 MTT Assay

The MTT test was used to investigate fruit extract cytotoxicity. To promote cell adhesion, 20,000 cells per well were seeded into a 96‐well plate with 100 μL of complete growth medium and incubated overnight at 37°C and 5% CO_2_ before receiving fruit extracts. On the following day, cells were exposed to increasing concentrations of fruit extracts (1%, 3.75%, 7.5%, 25%, 50%, and 100% v/v) from a stock solution of 10 mg/mL (w/v) to determine the half‐maximal inhibitory concentration (IC_50_) for each extract. After 24 h, 20 μL of MTT solution was added to each well, and the plate was incubated for 3 h under standard conditions. Thereafter, the plate was centrifuged for 10 min at 2000 rpm to remove unreacted MTT from the supernatant. After adding 100 μL of solubilization solution to each well, the plate was incubated at 37°C on a plate shaker for 1 h to dissolve formazan crystals. Absorbance was quantified at 570 nm utilizing a microplate reader. Cell viability was assessed as the percentage of absorbance compared to untreated controls, facilitating the estimation of the IC_50_ value for each plant extract. IC_50_ values were estimated in GraphPad Prism 9 Software and determined by non‐linear regression analysis, applying a three‐parameter logistic (3PL) model ([inhibitor] vs. response). Data were normalized to the untreated control, which was defined as 100% viability, prior to curve fitting. All experiments were conducted in triplicate and repeated three times to guarantee repeatability. The cytotoxicity was evaluated by measuring the % inhibition, determining the IC_50_, and calculating the selectivity index (SI).

### 2.4 HPLC‐ESI‐MS/MS Analysis

Delphinidin‐3,5‐diglucoside chloride and kaempferol‐3‐glucoside were purchased from PhytoLab (Vestenbergsgreuth, Germany). The remaining analytical standards were supplied by Sigma‐Aldrich (Milan, Italy). Individual stock solutions of each analyte, at a concentration of 1000 mg L^−1^, were prepared by dissolving pure standards in HPLC‐grade methanol. All solvents used were HPLC grade. Before HPLC analysis, all samples were filtered with a Phenex RC 4 mm 0.2 μm syringeless filter, Phenomenex (Castel Maggiore, BO, Italy).

HPLC‐ESI‐MS/MS analysis was carried out in an Agilent 1290 Infinity series and a Triple Quadrupole 6420 from Agilent Technology (Santa Clara, CA) with an electrospray ionization (ESI) source operating in negative and positive ionization modes (Mustafa et al. 2022). The separation of components was achieved on a Synergi Polar‐RP C18 analytical column (250 × 4.6 mm, 4 μm). The mobile phase was a mixture of (A) water and (B) methanol, both with 0.1% formic acid, at a flow rate of 0.8 mL min^−1^ in gradient elution mode. The injection volume was 2 μL, and the temperature of the column was 30°C. In the ionization source, the drying gas temperature was 350°C, the gas flow rate was 12 L/min, the nebulizer pressure was 55 psi, and the capillary voltage was set to 4000 V. Detection was done in the dynamic‐multiple reaction monitoring (dynamic‐MRM) mode, and the dynamic‐MRM peak areas were integrated for quantification. The HPLC–MS/MS method was validated by assessing linearity, limits of detection (LODs), limits of quantification (LOQs), repeatability, and specificity, as summarized in Table S1.

### 2.5 Statistical Analysis

Data were expressed as means ± standard deviation (SD), and a one‐way analysis of variance (ANOVA) was employed for multigroup comparison. Statistical analysis was carried out using GraphPad Prism. A *p*‐value < 0.05 was considered statistically significant.

## 3 Results

### 3.1 Antioxidant Activity

The evaluation of the antioxidant activity of the three extracts of *C. australis L*., carried out using the DPPH, ABTS, and FRAP methods, revealed significant free radical scavenging and ferric reducing capacities, depending on the extraction solvent (Table 1). Significant differences were found between the extraction solvents and in comparison with ascorbic acid. In the DPPH assay, ascorbic acid exhibited the highest antioxidant activity, with an IC_50_ value of 24.85 ± 1.28 μg/mL. In contrast, *C. australis* L. extracts showed higher IC_50_ values, indicating lower antioxidant activity. Among the extracts, the EtOH extract showed the lowest IC_50_ (110.53 ± 11.05 μg/mL), followed by the aqueous extract (409.60 ± 40.96 μg/mL) and MeOH extract (435.80 ± 43.58 μg/mL). Overall, these results demonstrate the value of organic solvents (MeOH and EtOH) for the effective extraction of antioxidants. Furthermore, the enhanced efficacy of extracts obtained with organic solvents highlights their ability to preferentially extract bioactive compounds responsible for antioxidant activity, especially those involved in iron reduction and radical scavenging. Finally, the complementarity of the methods used—DPPH (lipophilic radicals), ABTS (hydrophilic radicals), and FRAP (reducing capacity)—allows a more comprehensive evaluation of the antioxidant capacity of the extracts.

**TABLE 1:** In vitro evaluation of the antioxidant activity of the three extracts by DPPH, ABTS, and FRAP.

	DPPH (IC_50_ in μg/mL)	ABTS (IC_50_ in μg/mL)	FRAP (mg AAE/g of extract)
MeOH	435.80 ± 43.58^c^	151.88 ± 15.19^b^	5.73 ± 0.93^a^
EtOH	110.53 ± 11.05^b^	192.46 ± 19.25^c^	6.24 ± 1.21^a^
W	409.60 ± 40.96^c^	206.82 ± 20.68^c^	2.09 ± 1.09^c^
Ascorbic acid	24.85 ± 1.28^a^	60 ± 2.81^a^	—

*Note:* Values represent the means ± standard deviations of three assays; values with different superscript letters (a–c) are significantly different from each other with *p* < 0.05.

Abbreviation: AAE, ascorbic acid equivalent.

### 3.2 Cytotoxic Activity

#### 3.2.1 Determination of the % of Inhibition

**FIGURE 1:**
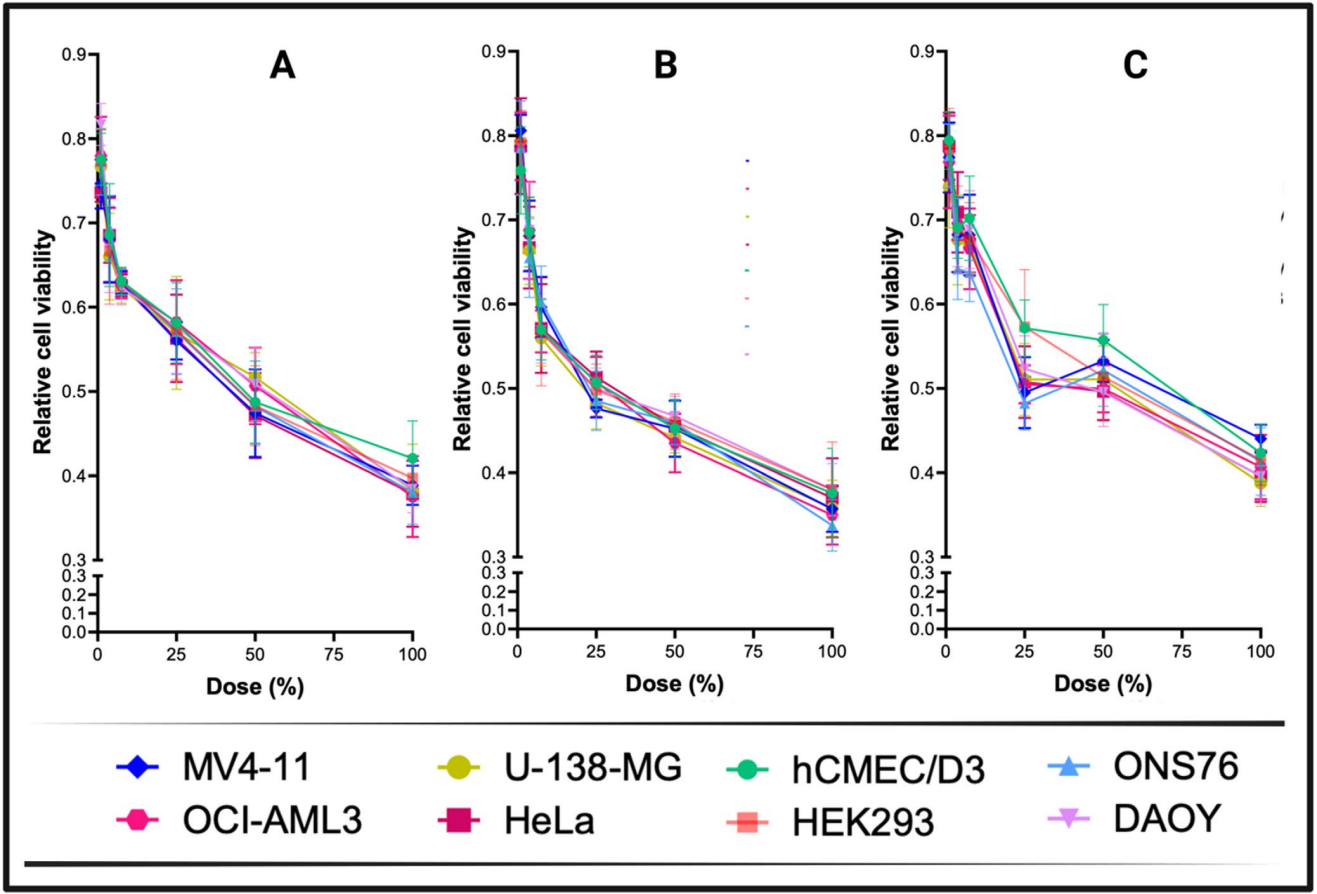
% of inhibition of cell viability by (A) MeOH, (B) EtOH, and (C) W extracts. Cells were treated with six increasing concentrations (1%, 3.75%, 7.5%, 25%, 50%, and 100% v/v) of *C. australis* L. extracts prepared using methanol (MeOH), ethanol (EtOH), and distilled water (W).

The viability of all cell lines that were tested displayed a dose‐dependent decrease (Table 2, Figure 1). The aqueous extract (W) was the most efficacious overall, particularly at higher doses, with DAOY exhibiting the highest inhibition (73.5% at a concentration of 100%). Aqueous extract maintained a consistent and high level of inhibition across all lines at a concentration of ≥ 25%. At intermediate concentrations (particularly 50%), the EtOH extract demonstrated marginally superior performance to MeOH. Its most potent effects were observed in OCI‐AML3 (67%), ONS76 (66.3%), and HeLa (63%) at a concentration of 100%, with DAOY being marginally less inhibited (62%). EtOH was particularly efficacious against medulloblastoma (ONS76) and leukemia (OCI‐AML3).

**TABLE 2:** Inhibition of cell viability (%) induced by MeOH, EtOH, and W extracts.

	% of extract	hCMEC/D3	HEK293	ONS‐76	DAOY	MV4‐11	OCI‐AML3	U‐138‐MG	HeLa
MeOH	1%	20.6	21.8	22.5	24	22.6	23.1	26.5	21.3
	3.75%	29.9	31.1	35.9	31.2	31.7	30.5	34.1	29.2
	7.5%	31	32.3	36.7	31.3	31.8	33.5	34.5	34.5
	25%	42.9	42.7	51.8	47.7	46.8	49.5	49	49.3
	50%	44.3	48.7	47.9	50.5	50.5	50.1	49	50.4
	100%	57.7	58.5	58.7	60.5	56	60	61.2	61.5
EtOH	1%	25	21.7	23.2	23.2	19.4	21.3	21	21.3
	3.75%	31.5	31.7	34.5	31.3	33.6	32	33.7	33
	7.5%	43.1	43.4	40	43.2	40.4	43.3	44	43
	25%	49.3	50.2	51.6	50	52.5	49.2	51.8	48.7
	50%	54.8	54	54.2	53.4	54.8	65.5	56	64.5
	100%	62.4	62	66.3	62	64.3	67	64	63
W	1%	21.7	23.6	21.4	22	22.2	33.2	21.8	22.5
	3.75%	34.2	33.8	35	33	33	34.3	33.1	33.2
	7.5%	48.5	51.4	50.8	49.3	49.3	51.7	48	48
	25%	56.2	58	58.5	57.5	56.9	58.7	56.1	54
	50%	61.7	62	62.5	61.4	61	61.7	61.5	61
	100%	62.8	63	63.8	73.5	63.3	63.8	62.3	63.2

The MeOH extract exhibited a consistent dose‐dependent increase in inhibition across all lines, with HeLa (61.5%), U‐138‐MG (61.2%), and OCI‐AML3 (60%) being the most responsive at a concentration of 100%. While glioblastoma U‐138‐MG and leukemia OCI‐AML3 also responded powerfully, DAOY and ONS76 medulloblastoma cells were highly sensitive, particularly to aqueous (W) and EtOH extracts. The inhibition of HeLa cells was consistent across all extracts. The aqueous extract exhibited moderate sensitivity in non‐cancer lines (HEK293, hCMEC/D3), with a slightly higher responsiveness, indicating limited selectivity.

In general, the aqueous extract exhibited the broadest and most potent activity, particularly in DAOY, whereas the EtOH extract was particularly efficacious in leukemia and medulloblastoma cells. Variations in solvent efficacy, compound solubility, and cell‐type sensitivity are likely the cause of these discrepancies.

#### 3.2.2 Determination of the IC_50_

To quantify cytotoxic potency, IC_50_ values, the concentration required to reduce cell viability by 50%, were determined for each extract and cell line (Table 3). Considering the IC_50_ values, EtOH showed consistently low IC_50_ values across all cell lines (range: 5.62%–10.01%), which make it the most potent extract in 4 out of 6 cancer cell lines (DAOY, MV4‐11, U‐138 MG, and HeLa), while W extract was extremely potent for OCI‐AML3 (3.66%) and OSN‐76 (6.79%) and weaker for hCMEC/D3 (36.16%) and HEK293T (31.15%), suggesting a cell‐type selectivity. MeOH extract was generally less potent (IC_50_ > 20% for all lines), especially in OCI‐AML3 (52.98%), U‐138 MG (50.41%), and HeLa (43.54%), with only moderate potency seen in DAOY (11.16%) cells. Overall, OCI‐AML3 (leukemia) is the most sensitive cell line, particularly to aqueous extract, while U‐138 MG and HeLa cells showed excellent responses to EtOH.

**TABLE 3:** IC_50_ values (%) of MeOH, EtOH, and W extracts on used cell lines (the lowest IC_50_ values indicating the highest cytotoxic activity are highlighted in bold).

Cell line	IC_50_ MeOH (%)	IC_50_ EtOH (%)	IC_50_ W (%)
hCMEC/D3	20.26 ± 1.21^**b**^	**9.07 ± 1.95** ^**b**^	36.16 ± 2.02^**f**^
HEK293T	26.80 ± 3.31^**b**^	**5.62 ± 0.67** ^**a**^	31.15 ± 2.84^**e**^
OSN‐76	33.57 ± 5.71^**c**^	10.01 ± 1.4^**b**^	**6.79 ± 0.61** ^**b**^
DAOY	11.16 ± 1.63^**a**^	**6.34 ± 0.87** ^**a**^	22.67 ± 0.60^**d**^
MV4‐11	33.75 ± 4.97^**a**^	**7.27 ± 2.35** ^**a**^	15.61 ± 0.47^**c**^
OCI‐AML3	52.98 ± 8.29^**d**^	8.49 ± 1.51^**b**^	**3.66 ± 0.47** ^**a**^
U‐138 MG	50.41 ± 6.97^**d**^	**5.71 ± 0.73** ^**a**^	29.14 ± 1.33^**e**^
HeLa	43.54 ± 5.31^**d**^	**6.47 ± 0.90** ^**a**^	15.83 ± 1.37^**c**^

*Note:* Values represent the means ± standard deviations of three assays; values with different superscript letters (a–f) are significantly different from each other with *p* < 0.05.

#### 3.2.3 Determination of Selectivity Index (SI)

**FIGURE 2:**
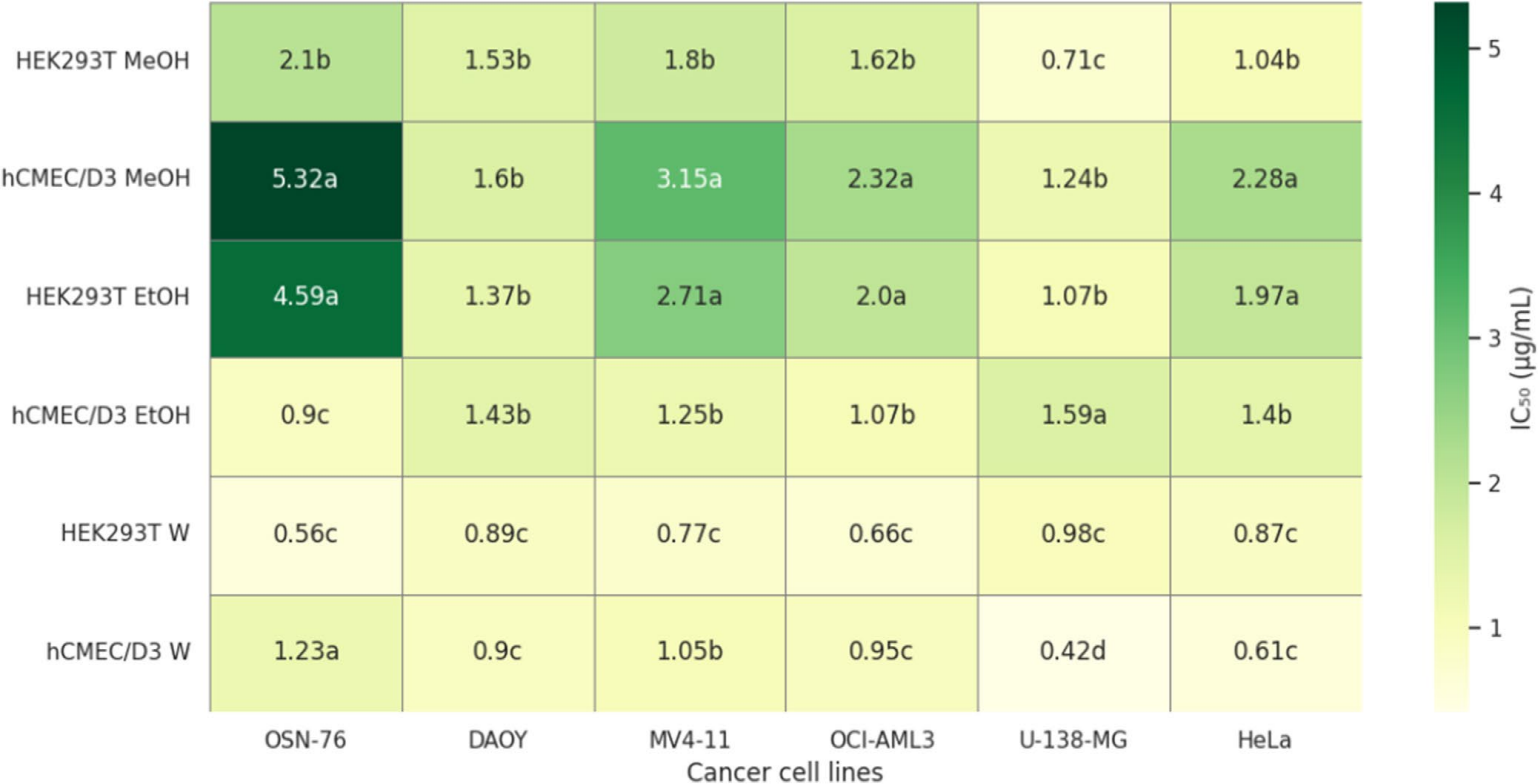
Heatmap showing the SI calculated against HEK293T and hCMEC/D3 cell lines. Values with different letters (a–d) are significantly different from each other with *p* < 0.05.

To assess the selectivity of the extracts, the SI was calculated by comparing IC_50_ values from cancer cell lines against those of non‐cancerous HEK293T and hCMEC/D3 cells (Figure 2). An SI > 1 indicates preferential toxicity toward cancer cells (i.e., the extract is more toxic to tumor cells than to normal cells—desirable for anticancer agents). Overall, MeOH and EtOH extracts exhibited significantly higher SI values, indicating higher selectivity, than W extract for most cancer cell lines, particularly against OSN‐76, MV4‐11, OCI‐AML3, and HeLa cells. For example, based on hCMEC/D3 result, MeOH extract showed significantly higher selectivity toward OSN‐76 (SI = 5.32) compared with EtOH (SI = 0.9) and W (SI = 1.23) extracts indicating a statistically supported enhancement of cancer selectivity. Similarly, based on HEK293T results, EtOH extract demonstrated significantly elevated SI values against OSN‐76 (SI = 4.59) and MV4‐11 (SI = 2.71). In contrast, W extract generally exhibited low SI values that were not statistically different from or were significantly lower than 1, suggesting limited or non‐selective cytotoxicity. This pattern was consistent across most cancer cell lines, particularly U‐138‐MG and HeLa cells, where SI values were < 1 and belonged to the lowest significance group. Indeed, in several cases, EtOH and MeOH extracts shared the same statistical grouping, indicating comparable selectivity rather than definitive superiority of one solvent system over another.

### 3.3 HPLC‐ESI‐MS/MS Analysis

The extraction yield varied depending on the solvent used. Water provided the highest yield (61.5 g/kg of dried fruit material, corresponding to 6.15%, w/w), followed by methanol (51 g/kg of dried fruit material, 5.10%, w/w), whereas ethanol resulted in a markedly lower yield (2 g/kg of dried fruit material, 0.20%, w/w) (Table 4). Both W and MeOH extracts were rich in hydroxycinnamic acids and flavonoid glycosides, namely neochlorogenic acid, procyanidin B2, (−)‐epicatechin, isoquercitrin, and delphinidin‐3,5‐diglucoside, accounting for 95% and 75% of the total, respectively. These are mostly flavonoids and proanthocyanidins, known for strong antioxidant and anti‐inflammatory activities. Phloretin was also detected in a good quantity in the W extract. Otherwise, chlorogenic acid, rutin, and kaempferol‐3‐glucoside were also detected in moderate levels in both extracts. MeOH extract was characterized by a poor overall yield but relatively higher neochlorogenic acid, delphinidin‐3,5‐diglucoside, and isoquercitrin, suggesting that ethanol helps to extract some mid‐polar compounds but less efficiently. Detailed quantities of the detected compounds and their structures are presented in Table 4. Our findings indicate that water and methanol, both highly polar solvents, are more efficient in extracting phenolic compounds from *C. australis* L. than ethanol. Hence, water and MeOH are favorable for polar biocompounds like procyanidins, epicatechin, and anthocyanins.

**TABLE 4:** Phenolic compounds detected in MeOH, EtOH, and water extracts of *C. australis* L. expressed in mg/kg.

Compounds	Standards characteristics							Quantification		
	Precursor ion, m/z	Product ion, m/z	Fragmentor, V	Collision energy, V	Polarity	Rt (min)	ΔRt	MeOH	EtOH	W
Gallic acid	169	125.2*	97	12	Negative	6.96	2	11.01	93.04	12.74
Neochlorogenic acid	353	191.2*, 179	82	12, 12	Negative	9.52	2	1994.82	411.23	2506.53
(+)‐Catechin	289	245.2*,109.2	131	8, 20	Negative	11.44	2	113.19	n.d.	32.24
Procyanidin B2	576.99	576.99*, 321.2	160	0, 32	Negative	12.41	2	12405.99	n.d.	14065.57
Chlorogenic acid	353	191.2*, 127.5	82	12, 20	Negative	12.42	2	581.39	294.21	505.89
*p*‐Hydroxybenzoic acid	137	93.2*	92	16	Negative	12.86	2	13.46	22.17	36.31
(−)‐Epicatechin	289	245.1*, 109.1	126	8, 20	Negative	13.03	2	10047.90	n.d.	13067.97
3‐Hydroxybenzoic acid	137	93.2*	88	8	Negative	13.59	2	n.d.	n.d.	n.d.
Caffeic acid	179	135.2*, 134.1	92	12, 24	Negative	13.65	2	6.23	n.d.	n.d.
Vanillic acid	167	152.4*, 108.1	88	12, 20	Negative	14.32	2	n.d.	n.d.	n.d.
Resveratrol	227	185*	131	12	Negative	14.40	2	n.d.	n.d.	n.d.
Syringic acid	196.9	182.2*, 121.2	93	8, 12	Negative	15.28	2	n.d.	n.d.	n.d.
Procyanidin A2	575	575*, 285	170	0, 20	Negative	16.18	2	n.d.	n.d.	n.d.
*p*‐Coumaric acid	163	119.2*, 93.2	83	12, 36	Negative	16.70	2	3.56	33.19	5.07
Ferulic acid	193	134.2*, 131.6	83	12, 8	Negative	17.10	2	n.d.	n.d.	n.d.
3,5‐Dicaffeoylquinic acid	514.9	353.1*, 191	117	8, 28	Negative	17.61	2	n.d.	n.d.	n.d.
Rutin	609	300.2*, 271.2	170	32, 50	Negative	17.73	2	614.27	17.70	494.71
Isoquercitrin	463	271.2*, 300.2	155	44, 24	Negative	18.36	2	12100.54	560.48	13929.48
Delphindin‐3,5‐diglucoside	462.9	300.1*	165	24	Negative	18.38	2	12702.11	615.62	15256.02
Phloridzin	435.39	273*, 167	155	8, 28	Negative	18.83	2	n.d.	7.30	41.60
Quercitrin	446.99	300.2*, 301.2	160	24, 16	Negative	19.61	2	n.d.	n.d.	n.d.
Myricetin	316.99	179.1*, 182	150	16, 24	Negative	19.61	2	n.d.	n.d.	n.d.
Naringin	578.99	271.3*, 151.3	170	32, 44	Negative	19.62	2	n.d.	n.d.	n.d.
Kaempferol‐3‐glucoside	447	284.2*, 255.2	170	24, 40	Negative	19.77	2	262.57	31.14	266.57
Ellagic acid	301	301*, 229	170	0, 24	Negative	21.41	2	77.83	n.d.	54.31
Quercetin	300.99	151.2*, 179.2	145	16, 12	Negative	21.87	2	51.21	6.67	41.94
Phloretin	272.99	167*, 123	116	8, 20	Negative	22.30	2	n.d.	n.d.	1197.9214
Isorhamnetin	314.99	300.2*, 196.1	145	16, 4	Negative	24.57	2	2.87	0.76	2.36
TOTAL								50,988.97	2093.53	61,517.24

*Note:* * These product ions were used for quantification.

Abbreviations: n.d., not detected; Rt, retention time; ΔRt, Delta retention time.

## 4 Discussion

*C. australis* L. is commonly used to treat many diseases like gastrointestinal problems, menstrual bleeding and amenorrhea (Touhtouh et al. 2025). The present study was designed to investigate the chemical constituents, antioxidant, and cytotoxic properties of MeOH, EtOH and aqueous extracts of Moroccan *C. australis* L. fruits. The antioxidant activity was evaluated by measuring their free radical scavenging potential, through the DPPH, FRAP and ABTS assays. Our results showed that *C. australis* L. has marked antioxidant potency corroborating those reported by Safari et al. (2023) and Filali‐Ansari et al. (2016). The antioxidant assays showed that *C. australis* L. extracts exhibited variable activity depending on the solvent. Methanolic and ethanolic extracts consistently demonstrated higher radical scavenging and reducing capacities than the aqueous extract, likely due to the poor solubility of active compounds in water. These finding align also with the study of Cascales and his team reporting variable FRAP values of 44.35–117.87 mg Fe^2+^/100 g (Cascales et al. 2021). Aditionally, when comparing our results with those reported by Cetiz and his team (Cetiz et al. 2024), both aqueous and 70% EtOH fruit extracts in their study exhibited markedly stronger antioxidant activity than those analyzed here, as reflected substantially lower IC_50_ values. In the DPPH assay, our extracts showed IC_50_ values of 110.53 ± 11.05 μg/mL (aqueous) and 409.60 ± 40.96 μg/mL (EtOH), whereas Cetiz et al. reported values of 10.22 ± 0.08 and 30.45 ± 0.17 μg/mL, respectively. A similar trend was observed in ABTS and FRAP assays (Cetiz et al. 2024). Overall, these comparisons indicate that the antioxidant capacity of our samples was considerably lower than that reported by Cetiz et al. likely reflecting differences in sample origin, extraction conditions, or phytochemical composition.

Beside, this study provides strong evidence for the cytotoxic activities of the fruits' extracts. Our findings corroborate those reported by El‐Alfy and his team, highlighting the promising efficacy of *C. australis* L. fruit extracts against other cancer cell lines (El‐Alfy et al. 2011). In fact, while studying the cytotoxic activity of ethanolic and aqueous extracts, HEP‐G2 (hepatocellular carcinoma) and NCI‐N87 (gastric carcinoma) cell lines were the most sensitive lines to both extracts in terms of ED50 (50% effective dose). COLO 205 (human colon carcinoma) cells were also very susceptible to ethanolic extract while it showed a moderate sensitivity to aqueous extract (El‐Alfy et al. 2011). Based on IC_50_ values, Cetiz and his team revealed that aqueous extract was more effective than 70% EtOH extract (Cetiz et al. 2024). Hence, the lower IC_50_ values were 31.13 and 39.97 μg/mL against HT‐29 (colorectal adenocarcinoma) and DU‐145 (prostate cancer) cell lines, respectively and a moderate activity was exhibited toward A549 (lung cancer), HGC‐27 (gastric cancer), HeLa (cervical cancer), and HCT‐116 (colorectal cancer) cell lines (Cetiz et al. 2024). Indeed, as we used the same HeLa cell line, our findings revealed that EtOH significantly inhibited its growth as compared to water extract which is in part contradictory to the findings of Cetiz and his team. Altogether, these findings emphasize the importance as well as the impact, both qualitative and quantitative, of choosing the best solvent to extract bioactive compounds (Do et al. 2014; Efferth and Kuete 2010; Stockert et al. 2012). Hence, the cytotoxic activity of plant extracts is highly dependent on the extraction solvent, as solvent polarity governs the chemical composition and cytotoxic potency of the recovered metabolites (Dai and Mumper 2010; Efferth and Kuete 2010). Recently, the ethanol extract of *C. australis* L. fruits showed a cytotoxic potency against the human ovarian cancer cell line A2780 (IC_50_ 251.43 μg/mL), while above a dose of 50 μg/mL, an increased the level of DNA damage was found, suggesting that *C. australis* L. mediates cell death by inducing DNA damage (Fidan et al. 2023). Moreover, our study revealed that *C. australis* L. fruit extracts exert a promising cytotoxic effect toward other cancer cell lines, including acute monocytic leukemia (MV4‐11 and OCI‐AML3), desmoplastic cerebellar medulloblastoma (DAOY), medulloblastoma (OSN‐76), and glioblastoma (U‐138‐MG) cell lines. EtOH fruit's extract were the most efficacious in terms of IC_50,_ not only as compared to W extract but also to MeOH extract. Moreover, beyond this comparative aspect, we substantially extends the anticancer relevance of *C. australis* L. by demonstrating, for the first time, its promising cytotoxic activity against a new panel of cancer cell lines. Notably, significant effects were observed against hematological malignancies, including acute monocytic leukemia (MV4‐11 and OCI‐AML3), as well as aggressive solid tumors such as medulloblastoma (DAOY and ONS‐76) and glioblastoma (U‐138‐MG).

A previous analysis of the chemical profile of *C. australis* L. ethanol and water fruit extract was conducted by Cetiz et al. by using the same technique, namely HPLC‐ESI‐MS/MS (Cetiz et al. 2024). As regards to the quantitative analysis, a total yield of 670.54 and 22.47 mg/kg were found in 70% EtOH and W extracts (Cetiz et al. 2024), which are far lower than the values obtained in our study with 2093.53 and 61517.24 mg/kg, respectively. Qualitatively, the study of Cetiz and his team revealed the presence of 11 compounds in the 70% EtOH extract, which were dominated by syringic acid (141 mg/kg), followed by neochlorogenic acid (134.85 mg/kg) and 4‐hydroxybenzoic acid (104 mg/kg), then catechin (80.79), caffeic acid (51.85), and low amounts of rutin, gallic acid, chlorogenic acid, and *p*‐coumaric acid. However, the water extract contained only two compounds in low content, namely neochlorogenic acid (16.81 mg/kg) and gallic acid (5.66 mg/kg). In comparison, our results revealed a substantially higher phenolic yield along with a broader diversity of compounds thereby redefining the phytochemical potential of this species.

Natural bioactive substances, especially dietary phenolic compounds, exhibit significant preventive and therapeutic effects against several forms of human cancer i.e., flavan‐3‐ols such as (−)‐epicatechin and (+)‐catechin, as well as for procyanidin B2 (Bakrim et al. 2022; Baranwal et al. 2022; Gopalakrishnan et al. 2018; Kiran et al. 2023; Li, Lu, et al. 2021; McMillan et al. 2007; Silva et al. 2025; Thomas and Dong 2021). Similarly, flavonols (e.g., rutin, isoquercitrin, isorhamnetin, and quercetin), phenolic acids (gallic, chlorogenic, caffeic, *p*‐coumaric, and ellagic acids), and dihydrochalcones (phloretin and phloridzin) have been reported to exhibit a marked antiumoral effect (Cai et al. 2020; Ceci et al. 2016; Dhyani et al. 2025; Fan et al. 2025; Hayakawa et al. 2020; Hou et al. 2017; Hoveizi and Hushmandi 2024; Hsiao et al. 2019; Kahkeshani et al. 2019; Li, Yu, et al. 2021; Liu et al. 2023; Nouri et al. 2020; Pandey et al. 2021; Pei et al. 2016; Sourani et al. 2016; Yousuf et al. 2020). Notably, both delphinidin‐3,5‐diglucoside and kaempferol‐3‐glucoside remain poorly characterized in terms of anticancer activity, highlighting the need for targeted studies to clarify their potential contribution. However, it should be emphasized that the present study does not allow direct attribution of the observed cytotoxicity to specific compounds, as their effective intracellular concentrations, and possible synergistic or antagonistic interactions within the extracts were not assessed.

To sum up, a very limited number of studies reported the cytotoxic effect of *Celtis* species against cancer cells. The current study not only enriches the phytochemical database of *C. australis* L. but also establishes a scientific basis for future anti‐cancer features of a *Celtis* species supporting the sustainable exploitation of this underutilized resource.

## 5 Conclusion

*C. australis* L. extracts exhibit dose‐dependent cytotoxicity against cancer cell lines and notable antioxidant properties in vitro. The extracts specifically hinder the viability of tumor cells while exerting little effect on normal cells. However, the results are limited to in vitro assays, and claims of selective toxicity and anticancer potential should be interpreted with caution. Further studies are necessary to evaluate the bioavailability, pharmacokinetics, metabolism, and in vivo toxicity of the active compounds, including isolating active molecules, understanding how they work at the molecular level, and testing their efficacy in living cancer models to determine their safety and therapeutic potential. These results emphasize the potential of the extracts to be further investigated as sources of bioactive compounds with anticancer properties.

## Author Contributions

**Ghazal Namazzadeh:** methodology, validation, writing – review and editing. **Tarik Aanniz:** conceptualization, methodology, supervision, writing – review and editing, data curation, software. **Mohammed Alorini:** methodology, writing – review and editing, visualization. **Khalil Hammani:** resources, project administration, supervision, writing – review and editing. **Giovanni Caprioli:** methodology, validation, writing – review and editing. **Abdelhakim Bouyahya:** investigation, writing – review and editing, methodology, validation. **Jihane Touhtouh:** methodology, writing – original draft, formal analysis, software, data curation. **Taoufiq Benali:** supervision, project administration, writing – review and editing. **Waleed Al Abdulmonem:** supervision, writing – review and editing, investigation, funding acquisition. **Laura Acquaticci:** methodology, writing – review and editing, validation.

## Funding

This work was supported by the Deanship of Graduate Studies and Scientific Research at Qassim University for financial support (QU‐APC‐2026).

## Conflicts of Interest

The authors declare no conflicts of interest.

## Supporting information


**Table S1:** Method validation data of the analyzed phenolic compounds by HPLC–MS/MS.

## Data Availability

The data that support the findings of this study are available from the corresponding author upon reasonable request.
