# Supplementary material for: Polyphenolic Diversity, Antioxidant Activity, and Anticancer Potential of Celtis australis L. Fruits: New Insights
Source: Food Sci Nutr. 2026 Apr 19;14(4):e71800. doi: 10.1002/fsn3.71800 (PMC13092726; doi:10.1002/fsn3.71800)
Supplement: Supplementary file 1 — Table S1: Method validation data of the analyzed phenolic compounds by HPLC–MS/MS. [file FSN3-14-e71800-s001.docx]

**SUPPLEMENTARY MATERIALS**

*Method validation*

The HPLC–MS/MS method was validated in terms of linearity, limits of detection (LODs), limits of quantification (LOQs), repeatability and specificity (**Table S1**). Calibration curves were constructed by injecting standard mixture solutions at the eight concentrations of 0.005, 0.01, 0.05, 0.1, 0.5, 1, 5 and 10 mg/L and the 28 analytes demonstrated good linearity (R^2^ ≥ 0.9943) in a wide concentration range. The LODs and LOQs were obtained by injecting serial dilutions of the corresponding standard solutions, taking the signal-to-noise (S/N) ratio of 3 and 10 as criteria, respectively. The signal-to-noise (SNR) ratio was measured using MassHunter Software from Agilent Technology (Santa Clara, CA). The LODs ranged from 0.0004 to 0.0033 mg/L, while the LOQs were defined in the range of 0.0012 to 0.01 mg/L, indicating an excellent sensitivity. The intraday precision (intraday repeatability or run-to-run precision) of the HPLC-MS/MS method was validated with the injection of the standard mixture solution under the selected optimal conditions five times a day. For interday precision (interday repeatability or day-to-day precision), measurements were conducted once a day on three consecutive days. All of the precision measurements were expressed as relative standard deviations (RSDs). The method revealed a very good precision with inter and intraday variations where RSD (%) ranged from 0.23 to 4.51 and 0.11 to 3.77, respectively. High specificity was obtained using HPLC-MS/MS working in dynamic-MRM mode. The method specificity was evaluated by measuring retention time stability and setting multiple pairs of precursor/product ions. Retention time stability for each molecule was studied three times over a period of 3 days and expressed by RSDs% which were in all cases ≤ 1.27%..

**Table S1.** Method validation data of the analysed phenolic compounds by HPLC–MS/MS

| No. | Compounds | Conc.  range (mg/L) | R^2 a^ | LOD  (mg/L)^b^ | LOQ  (mg/L)^c^ |
| --- | --- | --- | --- | --- | --- |
|  |  |  |  |  |  |
| 1 | Gallic acid | 0.005-10 | 0.9956 | 0.003 | 0.009 |
| 2 | Neochlorogenic acid | 0.005-10 | 0.9984 | 0.002 | 0.006 |
| 3 | (+)-Catechin | 0.005-10 | 0.9977 | 0.0026 | 0.008 |
| 4 | Procyanidin B2 | 0.005-10 | 0.9961 | 0.0033 | 0.01 |
| 5 | Chlorogenic acid | 0.005-10 | 0.9993 | 0.0033 | 0.01 |
| 6 | *p*-Hydroxybenzoic acid | 0.005-10 | 0.9980 | 0.0027 | 0.0083 |
| 7 | (-)-Epicatechin | 0.005-10 | 0.9977 | 0.0025 | 0.0075 |
| 8 | 3-Hydroxy benzoic acid | 0.005-10 | 0.9992 | 0.0024 | 0.0072 |
| 9 | Caffeic acid | 0.005-10 | 0.9985 | 0.0015 | 0.0045 |
| 10 | Vanillic acid | 0.005-10 | 0.9943 | 0.0033 | 0.01 |
| 11 | Resveratrol | 0.005-10 | 0.9979 | 0.0009 | 0.0027 |
| 12 | Syringic acid | 0.005-10 | 0.9997 | 0.0032 | 0.01 |
| 13 | Procyanidin A2 | 0.005-10 | 0.9998 | 0.0033 | 0.0099 |
| 14 | *P*-Coumaric acid | 0.005-10 | 0.9990 | 0.0031 | 0.0093 |
| 15 | Ferulic acid | 0.005-10 | 0.9959 | 0.0013 | 0.0039 |
| 16 | 3,5-Dicaffeoylquinic acid | 0.005-10 | 0.9999 | 0.0037 | 0.0111 |
| 17 | Rutin | 0.005-10 | 0.9985 | 0.003 | 0.009 |
| 18 | Isoquercitrin | 0.005-10 | 0.9999 | 0.0025 | 0.008 |
| 19 | Delphinidin-3,5-diglucoside | 0.005-10 | 0.9998 | 0.0019 | 0.006 |
| 20 | Phloridzin | 0.005-10 | 0.9995 | 0.0022 | 0.0066 |
| 21 | Quercitrin | 0.005-10 | 0.9990 | 0.003 | 0.009 |
| 22 | Myricetin | 0.005-10 | 0.9992 | 0.0026 | 0.008 |
| 23 | Naringin | 0.005-10 | 0.9970 | 0.0017 | 0.0051 |
| 24 | Kaempferol-3-glucoside | 0.005-10 | 0.9950 | 0.0016 | 0.005 |
| 25 | Ellagic acid | 0.005-10 | 0.9999 | 0.0033 | 0.01 |
| 26 | Quercetin | 0.005-10 | 0.9998 | 0.0015 | 0.0045 |
| 27 | Phloretin | 0.005-10 | 0.9951 | 0.0006 | 0.002 |
| 28 | Isorhamnetin | 0.005-10 | 0.9987 | 0.0004 | 0.0012 |

^a^ R^2^: Coefficient of determination

^b^ LODs: (limit of detection) = ratio of signal to noise (S/N) = 3

^c^ LOQs: (limit of quantification) = ratio of signal to noise (S/N) = 10
